# Supplementary material for: Gambling Environment Exposure Increases Temporal Discounting but Improves Model-Based Control in Regular Slot-Machine Gamblers
Source: Comput Psychiatr. 2022 Jul 5;6(1):142–65. doi: 10.5334/cpsy.84 (PMC11104401; doi:10.5334/cpsy.84)
Supplement: Supplemental Information. — Supplemental Tables, Figures and Results. [file cpsy-6-1-84-s1.pdf]

Supplemental Information

Supplemental Table S1. Baseline screening questionnaires.

	**Reference**	**Measure**
**AUDIT**	(Saunders et al., 1993)	Alcohol-Use-Disorders Identification Text
**BDI**	(Beck et al., 1996; Hautzinger et al., 2009)	Beck Depression Inventory II
**DSM-5**	(American Psychiatric Association, 2013; Falkai, 2015)	Diagnostic criteria for gambling disorder
**FTND**	(Heatherton et al., 1991)	Fagerström Test for Nicotine Dependence
**GRCS**	(Raylu & Oei, 2004)	Gambling-related cognition scale
**KFG**	(J. Petry & Baulig, 1996)	Kurzfragebogen zum Glücksspielverhalten
**SOGS**	(Lesieur & Blume, 1987)	South Oaks Gambling Screen

Supplemental Table S2. Summary of demographics and clinical information (n = 30).

	Mean	SD	Range
**Age**	30.87	7.94	20-47
**School years**	11.83	1.93	8-17
**Income**	1284.73	590.71	300-2500
**FTND**	2.5	3.01	0-9
**AUDIT**	7.6	7.10	0-26
**DSM-5**	5.9	2.02	3-9
**KFG**	27.54	10.31	7-54
**SOGS**	9.5	3.87	3-16
**BDI**	17.27	10.13	0-45
**GRCS**	17.60	4.54	9.57-30.4

Temporal discounting drift diffusion models (DDMs)

Model comparison and validation

We compared three versions of the drift diffusion model (DDM) that varied in the way that they accounted for the influence of value differences on trial-wise drift rates, based on model-fit (WAIC). To verify comparable model ranking across conditions, we first carried out a model comparison separately for each environment (see Supplemental Table S3). In both environments, a DDM with nonlinear drift-rate scaling (DDM_s) (Fontanesi et al., 2019; Peters & D'Esposito, 2020; Wagner et al., 2020) accounted for the data best when compared to a DDM with linear scaling (DDM_{lin}) (Pedersen et al., 2017) and a null model without value modulation (DDM_0).

We then build a full model with group level distributions for the baseline condition (neutral context) and s_x parameters for each model parameter x, modeling the change from the neutral to the gambling context. S_x parameters where modeled with Gaussian priors with means of zero (see methods section). Model ranking was confirmed for the full model (Supplemental Table S3). We next compared the DDMs and the softmax model with respect to the proportion of binary choices (LL vs. SS selections) that they correctly accounted for. As can be seen from Supplemental Table S4, the DDM_S and DDM_{lin} performed numerically on par with the softmax model, whereas the DDM_0 performed substantially worse (see

Supplemental Figure S1, Supplemental Table S4). Posterior predictive checks for the winning model showed that it accurately captured the effect of decision conflict (value difference) on RTs (see section *Posterior Predictive Checks* below and Supplemental Figure S2). Parameter recovery for this model was reported in our prior papers (Peters & D'Esposito, 2020; Wagner et al., 2020).

Supplemental Table S3 Temporal discounting DDM model comparison using the Watanabe-Akaike Information Criterion (WAIC) revealed the same model ranking for each context (neutral vs. gambling) and the full model. Scores are WAIC (SE).

	**Neutral**	**Gambling**	**Full model**
**DDM_0**	12037.7 (150.1)	11754.9 (157.7)	23792.4 (217.4)
**DDM_{lin}**	9304.6 (155.5)	9174.7 (158.5)	18949.6 (219.0)
**DDM_s**	8982.3 (155.6)	8744.6 (157.6)	17656.0 (220.8)

Supplemental Table S4 Proportions of correctly predicted binary choices (mean [range]) for the temporal discounting models (neutral vs. gambling context; see Supplemental Figure S1).

	**Neutral**	**Gambling**
**Softmax**	0.89 [0.57-0.98]	0.90 [0.66-1.00]
**DDM_0**	0.74 [0.52-0.93]	0.76 [0.55-0.99]
**DDM_{lin}**	0.89 [0.58-0.98]	0.90 [0.65-0.99]
**DDM_s**	0.90 [0.60-0.99]	0.91 [0.70-1.00]

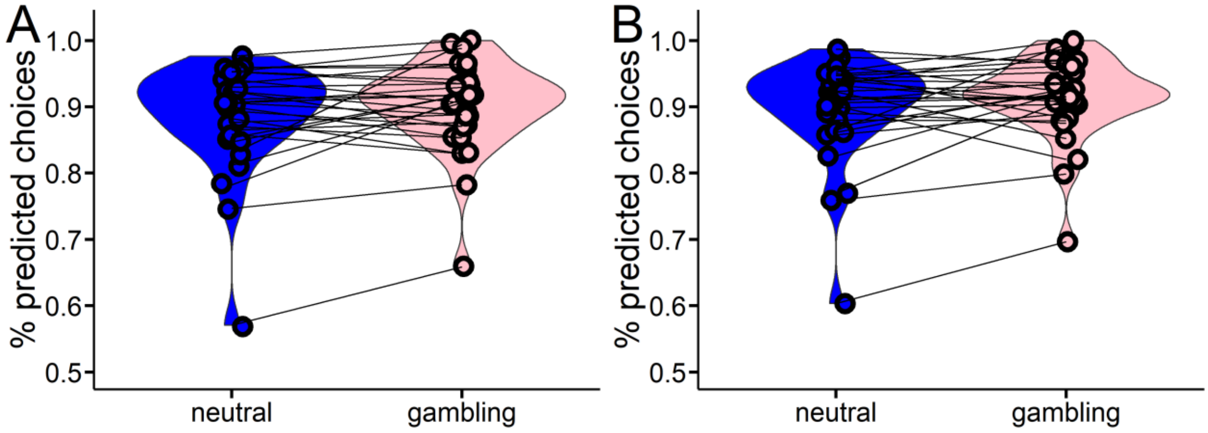

Supplemental Figure S1. Intertemporal choice task: Proportions of correctly predicted binary choices for the softmax model (A) and the drift diffusion model with non-linear drift rate scaling (B, DDMs) in both contexts (neutral [blue], gambling [pink]).

Posterior predictive checks

Temporal discounting

We carried out posterior predictive checks to visualize if our computational analysis captures key aspect in the data, in particular the value-dependency of RTs (Peters & D'Esposito, 2020; Wagner et al., 2020) . For the temporal discounting task, we binned trials per participant into five bins according to the absolute difference in larger-later vs. smaller-sooner value ("decision conflict", computed according to each participant's median posterior log(k) parameter from the DDM_S, and separately for the neutral and gambling context conditions). We then plotted the mean observed RTs as a function of decision conflict per participant and context, as well as the mean RTs across 10.000 data sets simulated from the posterior distributions of the DDM_0, DDM_{lin} and DDM_S (see Supplemental Figure S2).

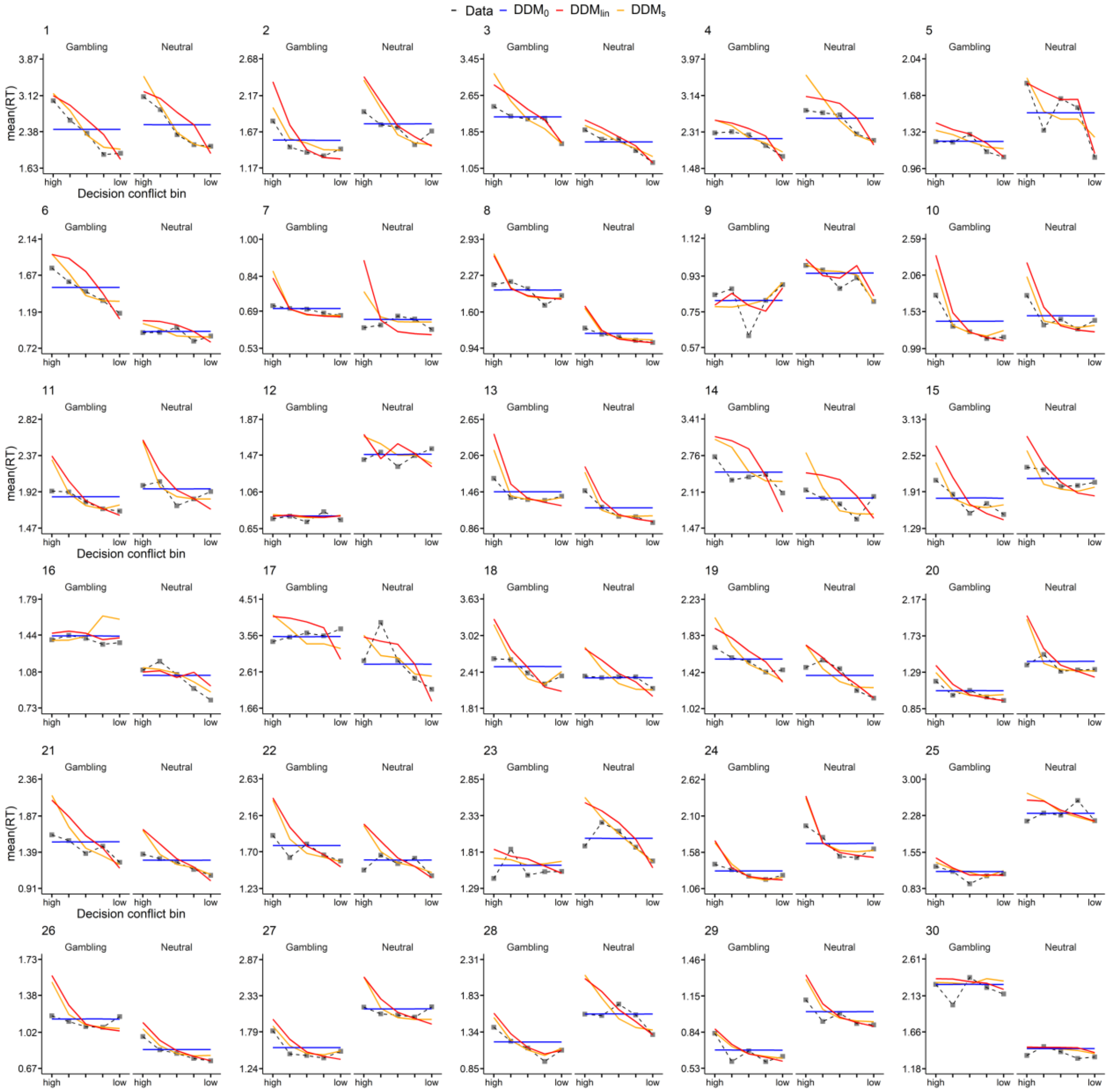

Supplemental Figure S2. Posterior predictive checks for temporal discounting drift diffusion models. For each participant and condition (Gambling vs. Neutral), trials were binned into five equal sized bins according to the absolute difference between subjective LL and SS option values (decision conflict bin). Plotted are mean observed RTs per bin (data) as well model-generated RTs (blue: DDM_0, red: DDM_{lin}, orange: DDM_S) averaged over 10,000 datasets simulated from the respective posterior distributions of the hierarchical models.

Supplemental Table S5. Model agnostic analysis of stay probability via a hierarchical general linear model (HGLM). HGLMs were estimated for each context separately using reward and transition as fixed and subject as random effects. The full model with stay probability as dependent variable included the predictors reward, transition (rare vs. common) and context (gambling vs. neutral) as fixed effects and subject as random effect. As a model-agnostic performance measure, the probability of choosing the same S1 option as in the previous trial (stay-probability) is typically analyzed as a function of reward, transition, and their interaction (Daw et al., 2011). Since the 2-step task version employed here utilized continuous payoffs, every trial was rewarded. The "reward" in S2 can thus not be used to directly predict stay probabilities, as done in previous work. Therefore, here the "reward" factor is computed relative to a moving average of recent rewards. Specifically, we categorized a reward R_{t-1} as positive "R+" if R_{t-1} was higher than the mean of last 7 rewards ($R_t > mean[R_{t-1:\,t-7}]$) and as negative "R-" if $R_t < mean(R_{t-1:t-7})$.

Neutral Context

	Estimate	z-Value	p
**Reward**	0.27350	4.127	3.68e-05***
**Transition**	0.29908	3.455	0.00055***
**Reward*Transition**	-0.51326	-4.190	2.79e-05***
**Gambling Context**			
**Reward**	0.38419	5.889	3.89e-09***
**Transition**	0.39439	4.653	3.27e-06***
**Reward*Transition**	-0.71324	-5.927	3.08e-09***
**Full Model**			
**Reward**	0.27361	4.131	3.60e-05***
**Transition**	0.29924	3.460	0.00054***
**Context**	-0.12701	-0.426	0.67015
**Reward*Transition**	-0.51377	-4.199	2.68e-05***
**Reward*Context**	0.11057	1.190	0.23423
**Transition*Context**	0.09502	0.785	0.43249
**Reward*Transition*Context**	-0.19892	-1.160	0.24614

Model free analysis of Stage 1 RTs

S1 RTs were modeled as a function of categorized reward in the previous trial (see previous section for how this was defined) and context as fixed effects and trial and subject as random effects. Previous reward significantly increased RTs ($t = -2.431$, $p = 0.015$, see Supplemental Table S6). We also observed a reward * context interaction (see Supplemental Table S6) on stage 1 RTs. RTs were slower following rewarded trials, more so in the gambling than the neutral context.

Supplemental Table S6. Hierarchical general linear model results of S1 RTs with reward and context as fixed effects and subject as random effect.

**S1 RT Model**			
	Estimate	t-Value	p
**Reward**	-0.025	-2.431	0.015*
**Context**	-0.0005	-0.044	0.97
**Reward*Context**	0.029	2.050	0.04*

Model free analysis of Stage 2 RTs

Supplemental Table S7. Model agnostic analysis of S2 RTs. HGLM with transition and context as fixed effects and subject as random effect.

**S2 RT Model**			
	**Estimate**	**T-statistic**	p
**Transition**	0.13	17.227	< 2e-16***
**Context**	0.0004	0.07	0.94
**Transition*Context**	0.02	1.804	0.07

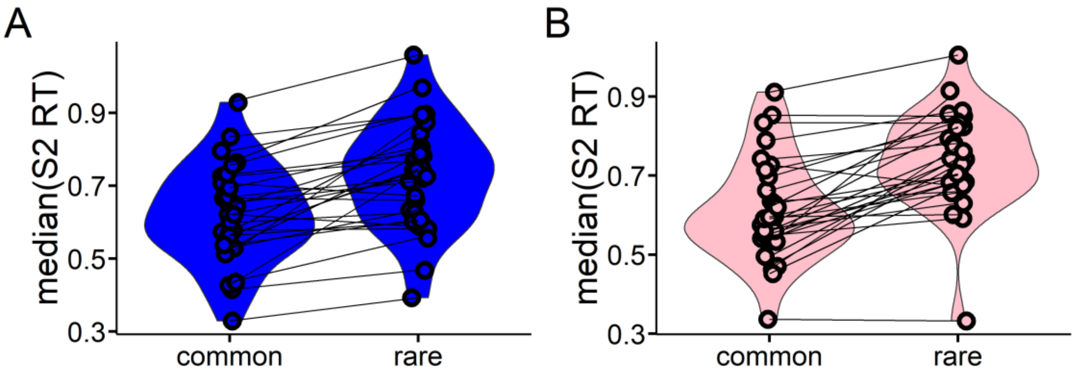

Supplemental Figure S3 Model free analysis of S2 RTs. RTs were substantially slower following rare transitions, both in the neutral (A) and the gambling context (B), see also Table 4.

Hybrid model with drift diffusion choice rule

Model comparison and validation

Model comparison based on the WAIC (Vehtari et al., 2017) (see Supplemental Table S8) revealed that in the neutral context, a DDM with nonlinear drift-rate scaling DDM_s (Fontanesi et al., 2019; Peters & D'Esposito, 2020; Wagner et al., 2020) accounted for the data best when compared to a DDM with linear drift rate scaling (DDM_{lin}) (Pedersen et al., 2017) and a null-model without learning (DDM0) (see Supplemental Table S8). The same ranking held for the gambling context.

We next build a full model with group level distributions for the baseline condition (neutral context) and additional s_x parameters for each model parameter x, modeling the change in from the neutral to the gambling context. These s_x parameters where modeled with Gaussian priors with means of zero (see methods section). The full model reproduced the model ranking (see Supplemental Table S8). We then compared the three DDMs and the softmax model with respect to the proportion of binary choices that they correctly accounted for. As can be seen from see Supplemental Table S9, the DDM_S and DDM_{lin} performed numerically on par with the softmax model, whereas the DDM_0 performed substantially worse. Posterior predictive checks showed that the final model accurately captured the effect of reward differences on second stage RTs and reproduced choice behavior (see Supplemental Figure S4 and S5 below).

Supplemental Table S8. Reinforcement learning DDM model comparison using the Widely-Applicable Information Criterion (WAIC) revealed the same model ranking for each condition (neutral or gambling context) as well as for the full model. Scores are WAIC (SE).

	**Neutral**	**Gambling**	**Full model**
**DDM_0**	11808.5 (1549.3)	10942.1 (1569.2)	22764.0 (2193.7)
**DDM_{lin}**	4616.2 (1897.0)	4429.0 (1729.9)	15519.7 (3984.4)
**DDM_s**	4357.2 (1935.1)	4197.2 (1749.7)	8800.3 (2670.0)

Supplemental Table S9. 2-step task models. Proportions of correctly predicted binary choices (mean [range]) for all models.

	Neutral		Gambling	
	Stage 1	Stage 2	Stage 1	Stage 2
**DDM_0**	0.63 [0.49-1.00]	0.62 [0.50-0.78]	0.56 [0.46-0.99]	0.63 [0.51-0.79]
**DDM_{lin}**	0.74 [0.51-1.00]	0.80 [0.53-0.96]	0.74 [0.50-0.99]	0.81 [0.59-0.95]
**DDM_s**	0.74 [0.49-1.00]	0.80 [0.55-0.96]	0.72 [0.47-0.99]	0.81 [0.59-0.95]
Softmax	0.72 [0.42-1.00]	0.79 [0.49-0.96]	0.72 [0.45-0.99]	0.81 [0.56-0.96]

Posterior predictive checks

We conducted posterior predictive checks to evaluate if our different hierarchical models capture both the relationship of RTs and reward differences and the relationship of reward differences and optimal choices. An optimal choice here is defined as a choice for the transition that leads to the random walk with highest payout (stage 1) or the highest payout (stage 2). To this end, we binned all trials into five bins, according to the absolute max(reward) differences in stage 2. For each reward difference bin we then plot the mean observed RTs, as well as the mean simulated RTs across 200 datasets simulated using 200 randomly sampled parameter estimates of the whole the posterior distributions of the DDM_0, DDM_{lin}, and DDM_S. We further show the mean observed optimal choices (max[reward]) vs. the mean simulated optimal choices given our parameter samples of the posterior distribution of each model. These results are shown in Supplemental Figures S4 and S5. As can be seen, the DDM_s outperformed the other models in capturing the relationship of reward differences and optimal choices (Supplemental Figure S4) and provided the best account of how RTs vary as a function of reward differences (Supplemental Figure S5).

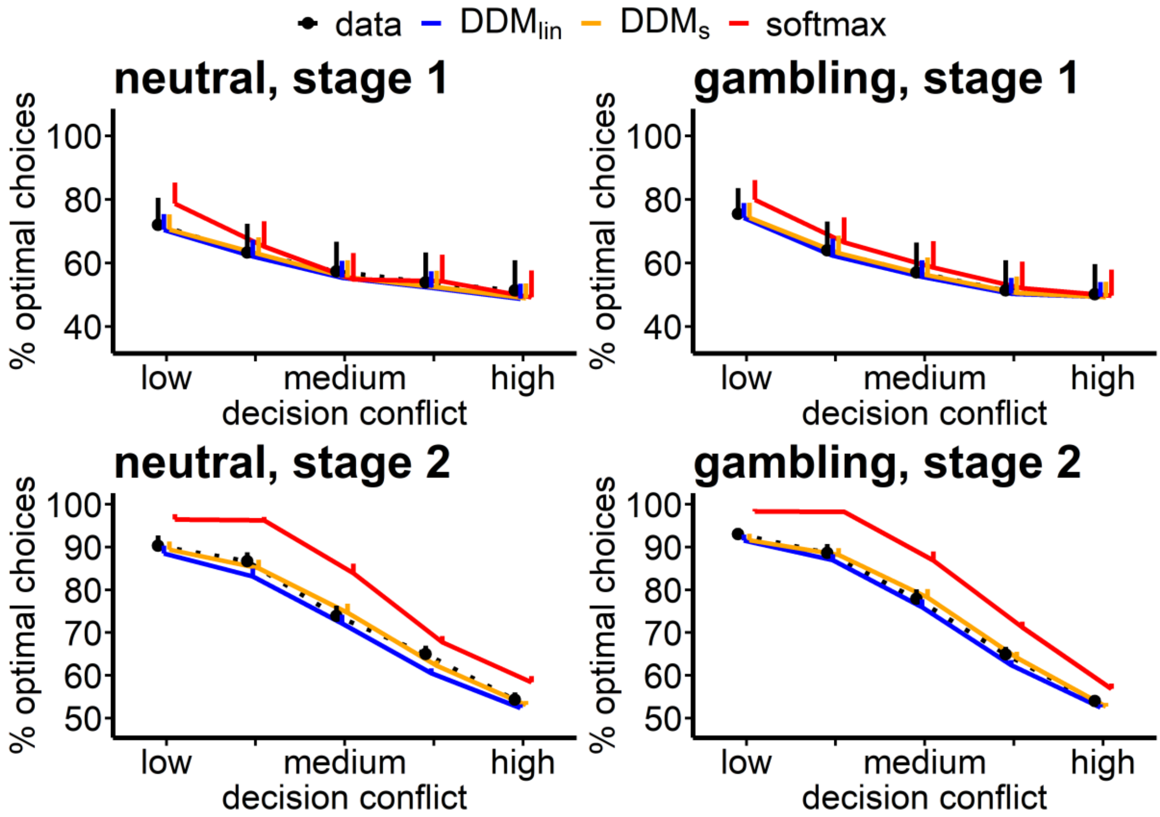

Supplemental Figure S4. Group level posterior predictive checks. Trials were binned into five equal sized bins (decision conflict). For S1 checks this refers to the absolute difference in max[S2 reward values (t-1)] between both S2 stages. In terms of S2 checks, decision conflict refers to the absolute S2 reward difference (t-1) within the current S2. Plotted are mean observed optimal choices per bin (data; dashed line) as well as model-generated choices (red: softmax model; blue: DDM_{lin}; orange: DDM_S) averaged over 200 datasets simulated from the whole posterior distribution of each hierarchical model.

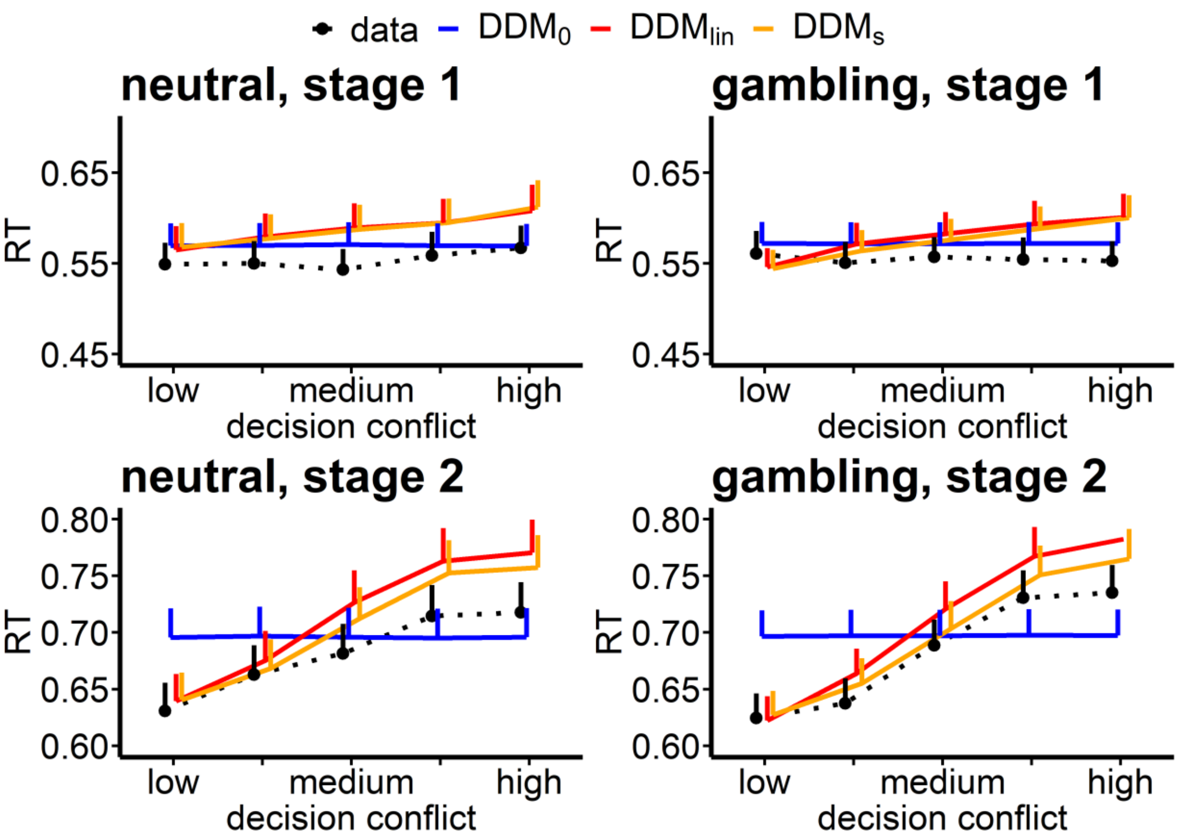

Supplemental Figure S5. Group level posterior predictive checks. Trials were binned into five equal sized bins (decision conflict). For S1 checks this refers to the absolute difference in max[S2 reward values (t-1)] between both S2 stages. In terms of S2 checks, decision conflict refers to the absolute S2 reward difference (t-1) within the current S2. Plotted are mean observed RTs per bin (data; dashed line) as well as model-generated RTs (blue: DDM_0; red: DDM_{lin}; orange: DDM_S) averaged over 200 datasets simulated from the whole posterior distribution of each hierarchical model.

Supplemental Results 1: Working memory, subjective craving and temporal discounting

Preregistered analysis:

We hypothesized a positive relationship of decision noise parameter and working memory z-score, which we confirmed in each context (neutral condition: $r = 0.45$, $p = 0.013$); gambling condition: $r = 0.45$ p = $p = 0.013$).

Exploratory analysis:

There was no significant correlation between discount-rate and working memory ($r = -0.03$, $p = 0.89$). A further exploratory analysis revealed an association of working memory and drift-rate coefficient. Here, higher working memory capacity was associated with higher drift rate coefficients (neutral condition: $r = 0.42$, $p = 0.02$; gambling condition: $r = 0.37$, $p = 0.048$). The difference in subjective craving from the neutral to the gambling environment, was not significantly associated with the change in the discount-rate (pre-task craving rating: $r = 0.28$, $p = 0.18$; post-task craving rating: 0.09, $p = 0.68$).

Supplemental Results 2: Working memory, subjective craving and 2-step task performance

Preregistered analysis:

WM z-score was positively but non-significant correlated with MB RL (neutral context: $r = 0.27$, $p = 0.16$, gambling context: $r = 0.31$, $p = 0.10$). The change in MB RL from neutral to gambling context was not significantly correlated with WM ($r = 0.27$, $p = 0.16$).

Exploratory analysis:

We further explored the association of WM capacity and S2 learning rates. This analysis revealed that overall WM capacity was positively associated with baseline (neutral context) ($r = 0.57$, $p = 0.001$) and gambling context ($r = 0.37$, $p = 0.048$) S2 learning rates. The change in subjective craving from the neutral to the gambling environment, was not significantly associated with the change in MB drift-rate weight (pre-task craving rating: -0.01, $p = 0.98$; post-task craving rating: $r = 0.33$, $p = 0.14$).

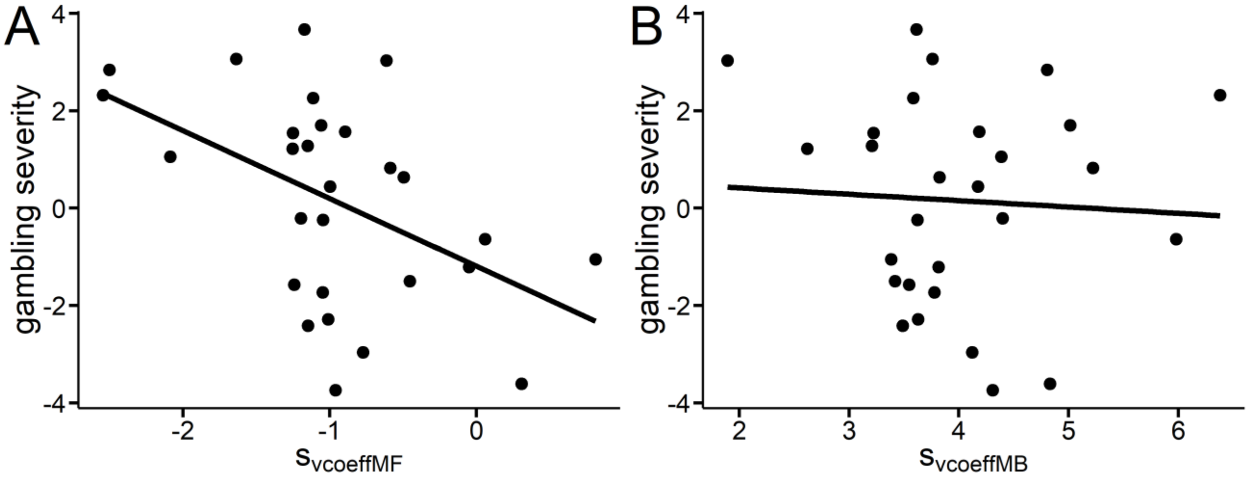

Supplemental Figure S6 Gambling symptom severity (y-axis; average z-score across DSM, KFG and SOGS) was associated with a greater gambling context related decrease in MF drift-rate weights ($S_{vcoeffMF}$, $r = -0.48$, $p = 0.009$). There was no association of Gambling symptom severity and the context related increase of MB drift-rate weights.

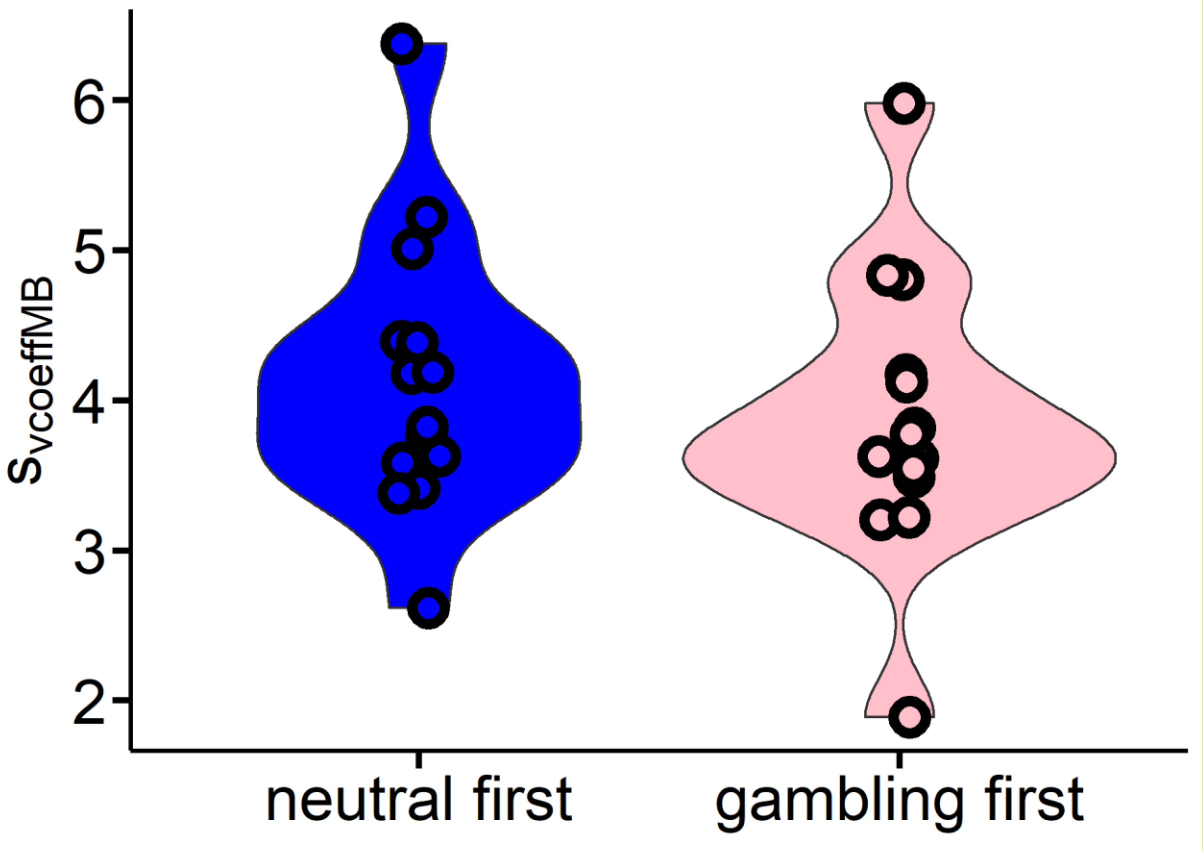

Supplemental Figure S7 Gambling context related shift in MB drift-rate weights as a function of order (neutral context first vs. gambling context first). Increases in MB drift-rate weights in the gambling context were observed in all participants regardless of order.

Additional analyses

We also examined an additional model in which perseveration was modeled as a change in starting point (bias) instead of change in drift rate. Posterior distributions for the perseveration parameter from this model are shown below. Note that in this revised formulation, there was no effect of perseveration on the starting point (see Supplemental Figure S8 below).

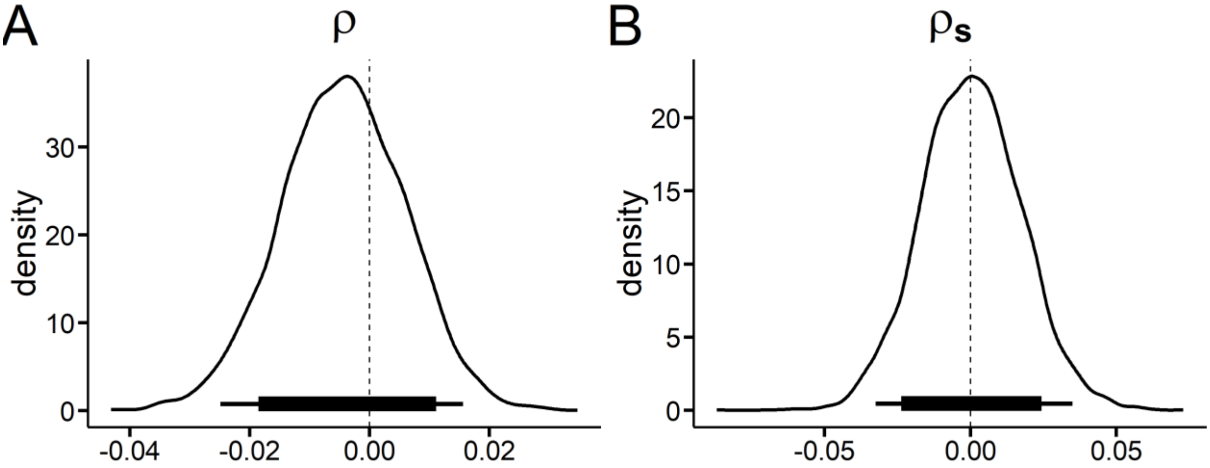

Supplemental Figure S8. A: Posterior hyperparameter distribution of perseveration when modeled as a change in starting point. B: Posterior hyperparameter distribution of the change in perseveration due to gambling context exposure.
